# Willingness to Continue Caregiving Among Informal Caregivers of Older Adults in Southwestern Nigeria

**DOI:** 10.1093/geroni/igaf122.2159

**Published:** 2025-12-31

**Authors:** Eniola Cadmus, Oluwakayode Ilugbo, Onyinye Mbanefo, Aderonke Alabi, Oluwaseun Ajayi, Eme Owoaje, Lawrence Adebusoye, Pamela Teaster

**Affiliations:** University of Ibadan, Ibadan, Oyo, Nigeria; University College Hospital, Ibadan, Oyo, Nigeria; Virginia Tech, Blacksburg, Virginia, United States; University College Hospital Ibadan, Ibadan, Oyo, Nigeria; University College Hospital, Ibadan, Oyo, Nigeria; University of Ibadan, Ibadan, Oyo, Nigeria; University of Ibadan, Ibadan, Oyo, Nigeria; Virginia Tech, Blacksburg, Virginia, United States

## Abstract

Informal caregiving of older persons is a time-consuming and demanding role often entered into with little preparation. Understanding the willingness of caregivers to continue in this role is essential for long-term care provision for older persons, especially in low-resource settings. This study assessed the willingness of informal caregivers to continue providing care for community-dwelling older persons in Oyo State, Southwestern Nigeria. A cross-sectional study was carried out using quantitative methods,, with data collected from 554 informal caregivers through a semi-structured, interviewer-administered questionnaire. The Caregiver Continuation Willingness Scale, Zarit Burden Interview, and a 15-item physical status assessment tool for older persons were used. Data were analyzed using SPSS version 25 at a significance level of p < 0.05. The caregivers had a mean age of 37.24 (±12.54) years, with most being female (83.6%), married (76.9%), and employed (93.5%). The older adults had a mean age of 79.81 (±12.28) years and were predominantly female (76.5%). Overall, 80.3% of caregivers expressed willingness to continue caregiving. Factors positively associated with willingness included being older, married, a close relative, experiencing mild caregiver burden, prolonged caregiving duration, emotional attachment, obligation to care, and caregiver satisfaction. Perceived ability to care (OR = 4.36; 95% CI [2.19, 8.68]; p < 0.001) and caregiver satisfaction (OR = 5.75; 95% CI [2.98, 11.08]; p < 0.001) were the strongest predictors of willingness to continue. These findings highlight the need for caregiver support programs and training to enhance caregiving skills and satisfaction, ultimately improving long-term care for older adults.

